# Creatinine, cystatin C, muscle mass, and mortality: Findings from a primary and replication population‐based cohort

**DOI:** 10.1002/jcsm.13511

**Published:** 2024-06-19

**Authors:** Dion Groothof, Naser B.N. Shehab, Nicole S. Erler, Adrian Post, Daan Kremer, Harmke A. Polinder‐Bos, Ron T. Gansevoort, Henk Groen, Robert A. Pol, Reinold O.B. Gans, Stephan J.L. Bakker

**Affiliations:** ^1^ Department of Internal Medicine, Division of Nephrology University Medical Center Groningen, University of Groningen Groningen the Netherlands; ^2^ Department of Biostatistics Erasmus Medical Center, Erasmus University Rotterdam Rotterdam the Netherlands; ^3^ Department of Epidemiology Erasmus Medical Center, Erasmus University Rotterdam Rotterdam the Netherlands; ^4^ Department of Internal Medicine Erasmus Medical Center, Erasmus University Rotterdam Rotterdam the Netherlands; ^5^ Department of Epidemiology University Medical Center Groningen, University of Groningen Groningen the Netherlands; ^6^ Department of Surgery, Division of Vascular and Transplantation Surgery University Medical Center Groningen, University of Groningen Groningen the Netherlands

**Keywords:** Creatinine, Cystatin C, General population, Kidney function, Mortality, Muscle mass

## Abstract

**Background:**

Serum creatinine is used as initial test to derive eGFR and confirmatory testing with serum cystatin C is recommended when creatinine‐based eGFR is considered less accurate due to deviant muscle mass. Low muscle mass is associated with increased risk of premature mortality. However, the associations of serum creatinine and cystatin C with muscle mass and mortality remain unclear and require further investigation to better inform clinical decision‐making.

**Methods:**

We included 8437 community‐dwelling adults enrolled in the Dutch PREVEND study and 5033 in the US NHANES replication cohort. Associations of serum creatinine and/or cystatin C with muscle mass surrogates and mortality were quantified with linear and Cox proportional hazards regression, respectively. Missing observations in covariates were multiply imputed using Substantive Model Compatible Fully Conditional Specification.

**Results:**

Mean (SD) age of PREVEND and NHANES participants (50% and 48% male) were 49.8 (12.6) and 48.7 (18.7) years, respectively. Median (Q1–Q3) serum creatinine and cystatin C were 71 (61–80) and 80 (62–88) μmol/L and 0.87 (0.78–0.98) and 0.91 (0.80–1.10) mg/L, respectively. Higher serum creatinine was associated with greater muscle mass, while serum cystatin C was not associated with muscle mass. Adjusting both markers for each other strengthened the positive relationship between serum creatinine and muscle mass and revealed an inverse association between serum cystatin C and muscle mass. In the PREVEND cohort, 1636 (19%) deaths were registered over a median follow‐up of 12.9 (5.8–16.3) years with a 10‐year mortality rate (95% CI) of 7.6% (7.1–8.2%). In the NHANES, 1273 (25%) deaths were registered over a median follow‐up of 17.9 (17.3–18.5) years with a 10‐year mortality rate of 13.8% (12.8–14.7%). Both markers were associated with increased mortality. Notably, when adjusted for each other, higher serum creatinine was associated with decreased mortality, while the association between serum cystatin C and increased mortality strengthened. The shapes of the associations in the PREVEND study and NHANES were almost identical.

**Conclusions:**

The strong association between serum creatinine and muscle mass challenges its reliability as GFR marker, necessitating a more cautious approach in its clinical use. The minimal association between serum cystatin C and muscle mass supports its increased use as a more reliable alternative in routine clinical practice.

## Introduction

The current guideline for evaluating and managing CKD recommends creatinine measures as initial test and an estimating equation to derive eGFR.^1^ Because approximately 98% of creatinine originates from muscle,^2^ circulating levels are positively related to muscle mass aside from their inverse relationship with GFR.^3^ Estimating equations include age and sex as variables to account for muscle mass‐related variability in creatinine.^4^ However, they do not account for settings with atypical muscle mass and the inverse relationships between kidney function and muscle mass.^5^, ^6^, ^7^ We recently showed this may bias creatinine‐based eGFR (eGFRcr) to clinically unacceptable levels,^7^ challenging the validity of creatinine as prime GFR marker in complex clinical settings.

Typically, patients with multimorbidity often have impaired kidney function and low muscle mass.^8^ This association can be partly explained by the spontaneous, nonenzymatic conversion of creatine to creatinine.^9^ Creatine, as a crucial growth factor for muscle‐protein synthesis,^6^ must be continually replenished to maintain muscle mass. Endogenous creatine production links impaired kidney function to decreased muscle mass,^5^ as the rate‐limiting step (i.e., guanidinoacetate production) occurs in the kidneys.^9^, ^10^ Ideally, filtration markers should be related to GFR solely through kidney clearance. Unlike cystatin C, which meets this criterion due to its inverse relationship with GFR^3^ and independence of muscle mass,^11^ creatinine clearly contradicts this principle.

Hypothetically, adjusting for cystatin C strengthens the positive association between circulating creatinine and muscle mass by detaching creatinine from its intrinsic relationship with kidney function. This principle also applies to the association between higher circulating creatinine and increased risk of adverse outcomes. While circulating creatinine is widely recognized as GFR marker,^3^ it primarily reflects muscle mass in dialysis patients who have essentially zero kidney function.^12^ This explains why low rather than high circulating creatinine is associated with excess mortality in these patients.^13^, ^14^ To disentangle these complex relationships and better inform clinical decisions on marker reliability in varying settings, we investigated whether cystatin C adjustment in a primary and replication population‐based cohort could clarify the associations of serum creatinine with muscle mass and mortality.

## Methods

### Study population and design

In this observational study, analyses were based on a primary cohort from the Prevention of REnal and Vascular ENd‐stage Disease (PREVEND) study and an independent replication cohort from the 2001–2002 U.S. National Health and Nutrition Examination Survey (NHANES) cycle. Full study details and flowcharts describing participant selection are provided in Data S1 study methods. Briefly, we included 8437 adults with available data on 24‐h creatinine excretion rate (CER) and mortality from the first screening of the PREVEND study. The replication cohort consisted of 5033 adults with available data on education, dual‐energy X‐ray absorptiometry (DXA), and mortality. The PREVEND study has been approved by the local medical ethics committee (MEC 96/01/022) and was undertaken in accordance with the Declaration of Helsinki. The National Health Statistics Research Ethics Review Board approved all NHANES protocols (Protocol #98‐12). All participants gave informed consent.

### Assessment and definitions of covariates and co‐morbidities

Covariates comprised potential confounders of the associations of serum creatinine and cystatin C with muscle mass as well as common risk factors for co‐morbidities and mortality: age, alcohol consumption, history of cardiovascular disease, malignancy, prevalent type 2 diabetes mellitus, serum creatinine and cystatin C, sex, smoking, and waist circumference. In the primary cohort, urinary albumin excretion was additionally considered. In the primary cohort, total‐body skeletal muscle mass was approximated using the equation 18.9 × CER + 4.1, with CER expressed in grams of urinary creatinine excreted in 24 h.^15^ As a second surrogate of muscle mass, the CER was indexed by height as previously described.^7^ This height‐indexed CER is hereafter referred to as ‘CER index’. In the replication cohort, total‐body skeletal muscle mass was approximated using DXA‐derived appendicular lean soft tissue (ALST) using the equation 1.19 × ALST − 1.01, with ALST referring to the sum of the upper and lower extremity lean soft tissue expressed in kilogram.^16^ Further information on definitions is provided in Data S1 study methods.

### Statistical analyses

To reduce potential bias due to missing data,^17^ multiple imputation of incomplete covariates using Substantive Model Compatible Fully Conditional Specification^18^ was performed to obtain multiple complete data sets. Analyses were performed in each data set and results were pooled using Rubin's rules (details are provided in Data S1 study methods).^19^, ^20^ Clinical characteristics are shown for complete case data (*n* = 7592 in PREVEND; *n* = 4339 in NHANES) and are expressed as mean (SD), median (Q1–Q3), or counts (percentage) for normally distributed, skewed, and categorical data, respectively. Imputed data were used in all subsequent analyses. In all analyses, serum creatinine, cystatin C, and urinary albumin excretion were log_2_‐transformed to stabilize variance and obtain approximately normal distributions.

Baseline effects of serum creatinine and cystatin C on muscle mass surrogates were quantified using linear regression models, specifying sex, current smoking, alcohol consumption, prevalent malignancy, prevalent type 2 diabetes mellitus, history of cardiovascular disease, and nonlinear effects of serum creatinine, serum cystatin C, age, waist circumference, and urinary albumin excretion. Details on these models are provided in Data S1 study methods. The resulting point estimates, confidence intervals, and *P* values are presented in Table S1. Moreover, results are visualized to facilitate their interpretation.

Median (Q1–Q3) follow‐up times were quantified with the reverse Kaplan–Meier method.^21^ Effects of serum creatinine and cystatin C on the hazard of death were quantified with Cox proportional hazards models, including sex, current smoking, alcohol consumption, prevalent malignancy, prevalent type 2 diabetes, and nonlinear effects of serum creatinine, serum cystatin C, age, waist circumference, and urinary albumin excretion as predictors. Two potential interactions were explored by introducing product terms of age with sex and waist circumference. Details on these models are provided in Data S1 study methods. Point estimates, confidence intervals, and *P* values are presented in Table S2. The results are visualized to facilitate their interpretation. Statistical analyses were performed with R version 4.3.3 (Vienna, Austria). A two‐sided *P* < 0.05 was considered to indicate statistical significance.

## Results

### Baseline characteristics

Clinical characteristics in the primary and replication cohort were very similar (Table 1). In the primary cohort, mean (SD) age of the 7592 participants (50% male) was 49.8 (12.6) years. Overall mean CER index was 7.2 (1.9) mmol/24 hour per meter and 8.2 (1.8) and 6.2 (1.3) mmol/24 hour per meter in males and females, respectively. This corresponded to an expected overall total‐body skeletal muscle mass of 30.3 (7.5) kg and 34.9 (6.9) and 25.7 (4.8) kg in males and females, respectively. Median (Q1–Q3) serum creatinine and cystatin C were 71 (61–80) μmol/L and 0.87 (0.78–0.98) mg/L, respectively. In the replication cohort, mean age of the 4339 participants (48% male) was 48.7 (18.7) years. Mean total‐body skeletal muscle mass was 26.0 (7.4) kg in the overall cohort and 30.9 (6.2) and 21.0 (4.8) kg in males and females, respectively. Median serum creatinine and cystatin C were 80 (62–88) μmol/L and 0.91 (0.80–1.10) mg/L, respectively.

**Table 1:** Clinical characteristics of complete case data at baseline and number of missing observations

Characteristic	Primary cohort (*n* = 7592)		Replication cohort (*n* = 4339)	
	Value	Missing (%)	Value	Missing (%)
Sociodemographic characteristics				
Age, mean (SD), y	49.8 (12.6)	0 (0.0)	48.7 (18.7)	0 (0.0)
Male sex, no. (%)	3787 (50)	0 (0.0)	2075 (48)	0 (0.0)
Race, no. (%)		0 (0.0)		0 (0.0)
Caucasian	7266 (96)		2335 (54)	
Black	72 (0.9)		763 (18)	
Other	254 (3)		1241 (29)	
Education, no. (%)		0 (0.0)		0 (0.0)
Low	3400 (45)		1247 (29)	
Middle	1924 (25)		1025 (24)	
High	2268 (30)		2065 (48)	
Smoking behaviour, no. (%)	2575 (34)	30 (0.4)	2115 (49)	592 (10)
Alcohol consumption, no. (%)	1921 (25)	43 (0.5)	2858 (69)	1370 (23)
Prevalent type 2 diabetes, no. (%)	265 (4)	0 (0.0)	465 (11)	697 (12)
Prevalent cardiovascular disease, no. (%)	385 (5)	0 (0.0)	439 (10)	582 (10)
Prevalent cancer, no. (%)	116 (2)	0 (0.0)	390 (9)	589 (10)
Body composition				
Height‐indexed CER, mean (SD), mmol/24 h per meter	7.0 (1.8)	0 (0.0)	‐	‐
Males	8.0 (1.7)	0 (0.0)	‐	‐
Females	6.1 (1.3)	0 (0.0)	‐	‐
Total‐body skeletal muscle mass, mean (SD), kg^a^	30.3 (7.5)	0 (0.0)	26.0 (7.4)	0 (0.0)
Males	34.9 (6.9)	0 (0.0)	30.9 (6.2)	0 (0.0)
Females	25.7 (4.8)	0 (0.0)	21.0 (4.8)	0 (0.0)
Waist circumference, mean (SD), cm	88.5 (13.0)	9 (0.1)	96.8 (15.1)	714 (12)
Males	93.8 (11.1)	3 (0.0)	99.3 (14.3)	310 (11)
Females	83.2 (12.7)	6 (0.1)	94.5 (15.4)	404 (13)
Hemodynamics				
Systolic blood pressure, mean (SD), mmHg	129 (20)	3 (0.04)	125 (20)	698 (12)
Diastolic blood pressure, mean (SD), mmHg	74 (10)	3 (0.04)	71 (14)	698 (12)
Lipid spectrum				
Total cholesterol, mean (SD), mmol/L	5.6 (1.1)	45 (0.54)	5.3 (1.1)	784 (13)
High‐density lipoprotein cholesterol, mean (SD), mmol/L	1.3 (0.4)	191 (2)	1.4 (0.4)	784 (13)
Total cholesterol/high‐density lipoprotein cholesterol ratio, mean (SD)	4.7 (1.8)	218 (3)	4.2 (1.5)	784 (13)
Triglycerides, median (Q1–Q3), mmol/L	1.16 (0.84–1.68)	190 (2)	1.38 (0.94–2.04)^b^	3393 (57)
Kidney function parameters				
Serum creatinine, median (Q1–Q3), μmol/L	71 (61–80)	532 (6)	80 (62–88)	775 (13)
Serum cystatin C, median (Q1–Q3), mg/L	0.87 (0.78–0.98)	534 (6)	0.91 (0.80–1.10)^c^	3472 (58)
Urinary albumin excretion, median (Q1–Q3), mg/24 h	9.4 (6.3–17.7)	0 (0.0)	‐	‐
Categories of urinary albumin excretion, no. (%), mg/24 h		0 (0.0)		‐
<15	5341 (70)		‐	‐
15–29.9	1116 (15)		‐	‐
30–300	1019 (13)		‐	‐
>300	116 (2)		‐	‐
**Medication**				
Antihypertensive drugs, no. (%)	1177 (16)	40 (0.48)	‐	‐
Lipid‐lowering drugs, no. (%)	485 (6)	33 (0.40)	‐	‐

Percentages may not total 100 because of rounding. Percentages greater than 1% were rounded to the nearest integer. A dash (−) means that no information on the specific variable was available.

CER, creatinine excretion rate; DXA, dual‐energy X‐ray absorptiometry.

^a^ In the replication cohort, mean and SD of total‐body skeletal muscle mass are pooled estimates over the initial five multiply imputed data sets as provided on the NHANES website (https://wwwn.cdc.gov/Nchs/Nhanes/Dxa/Dxa.aspx).

^b^ Data on triglycerides was available in 2183 of the 4339 complete cases.

^c^ Data on serum cystatin C was available in 2175 of the 4339 complete cases.

### Baseline effects of serum creatinine and cystatin C on muscle mass with and without adjustment for each other

**Figure 1:**
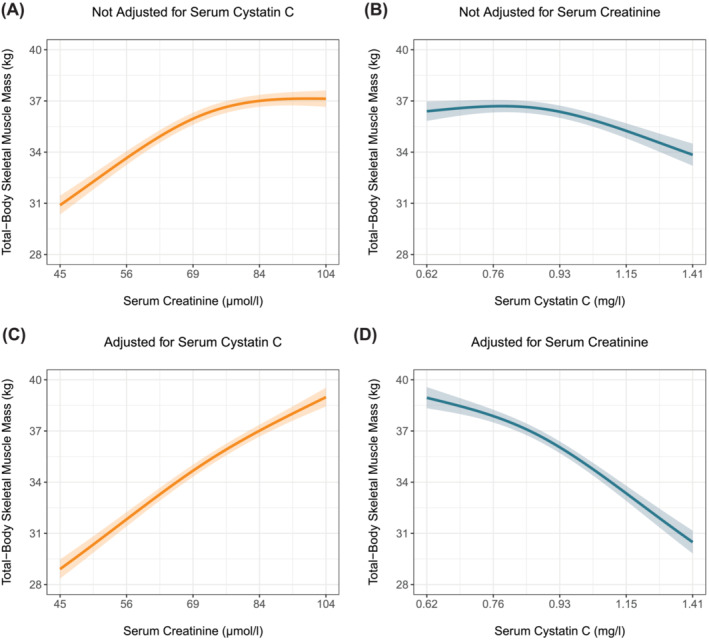
Associations of serum creatinine and cystatin C with total‐body skeletal muscle mass with and without adjustment for each other in the primary cohort. Expected total‐body skeletal muscle mass and associated 95% pointwise confidence intervals were derived from linear regression models and were (aside from serum creatinine and/or cystatin C) adjusted for the effects of age, sex, current smoking, alcohol consumption, prevalent malignancy, prevalent type 2 diabetes, history of cardiovascular disease, waist circumference, and urinary albumin excretion.

**Figure 2:**
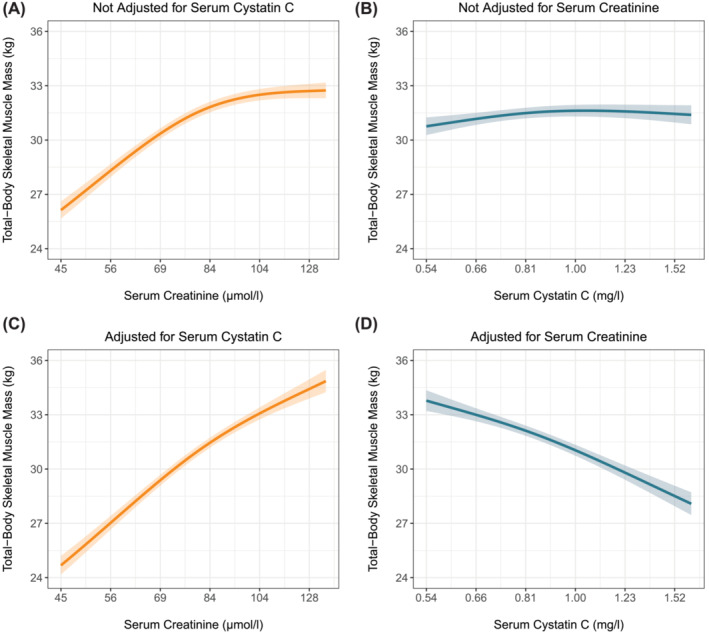
Associations of serum creatinine and cystatin C with total‐body skeletal muscle mass with and without adjustment for each other in the replication cohort. Expected total‐body skeletal muscle mass and associated 95% pointwise confidence intervals were derived from linear regression models and were (aside from serum creatinine and/or cystatin C) adjusted for the effects of age, sex, current smoking, alcohol consumption, history of malignancy, prevalent type 2 diabetes, history of cardiovascular disease, and waist circumference.

In the primary cohort, higher serum creatinine levels were associated with higher total‐body skeletal muscle mass (Figure 1A), whereas higher serum cystatin C levels were only modestly associated with lower total‐body skeletal muscle mass (Figure 1B); see also Table S1. Considering both markers together strengthened both the positive association between serum creatinine and total‐body skeletal muscle mass (Figure 1C) and the inverse association between serum cystatin C and total‐body skeletal muscle mass (Figure 1D). Notably, the shapes of the associations between serum creatinine and cystatin C with CER index were practically identical, whether the kidney function markers were considered individually or jointly (Figure S3A–D); see also Table S1. In the replication cohort, higher serum creatinine levels were associated with higher total‐body skeletal muscle mass (Figure 2A), whereas there was no association between higher serum cystatin C levels and total‐body skeletal muscle mass (Figure 2B). Considering both markers together strengthened the positive association between serum creatinine and total‐body skeletal muscle mass (Figure 2C) and revealed an inverse association between serum cystatin C and total‐body skeletal muscle mass (Figure 2D). Notably, the shapes of the associations in the primary and replication cohort were almost identical.

### Effects of serum creatinine and cystatin C on mortality with and without adjustment for each other

**Figure 3:**
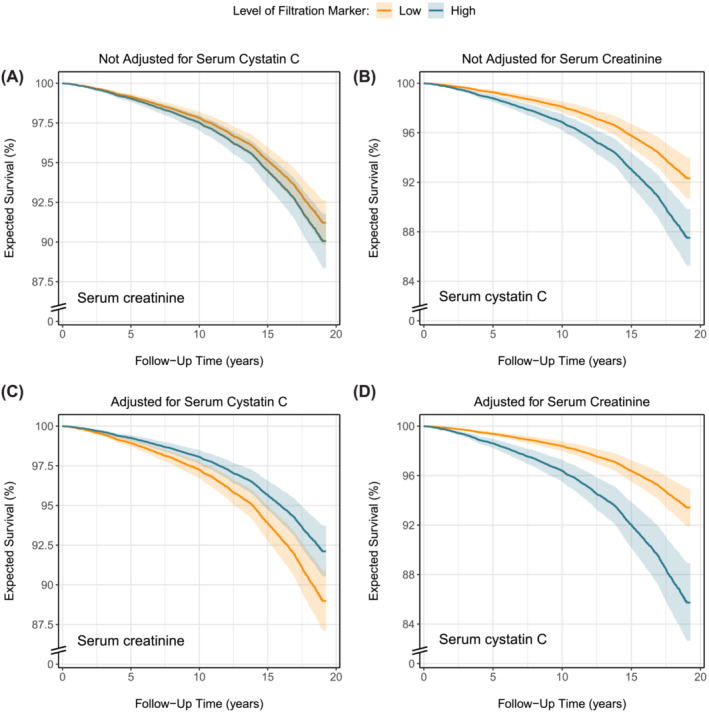
Effects of serum creatinine and cystatin C on the expected probability of survival with and without adjustment for each other in the primary cohort. Expected survival probabilities and associated 95% pointwise confidence intervals were derived from Cox models and were (aside from serum creatinine and/or cystatin C) adjusted for the baseline effects of age, sex, current smoking, alcohol consumption, prevalent malignancy, prevalent type 2 diabetes, history of cardiovascular disease, waist circumference, and urinary albumin excretion. Low and high levels of the filtration markers in the figure legend corresponded to the 2.5th and 97.5th percentile, which were 57 and 112 μmol/L for serum creatinine and 0.67 and 1.35 mg/L for serum cystatin C, respectively.

**Figure 4:**
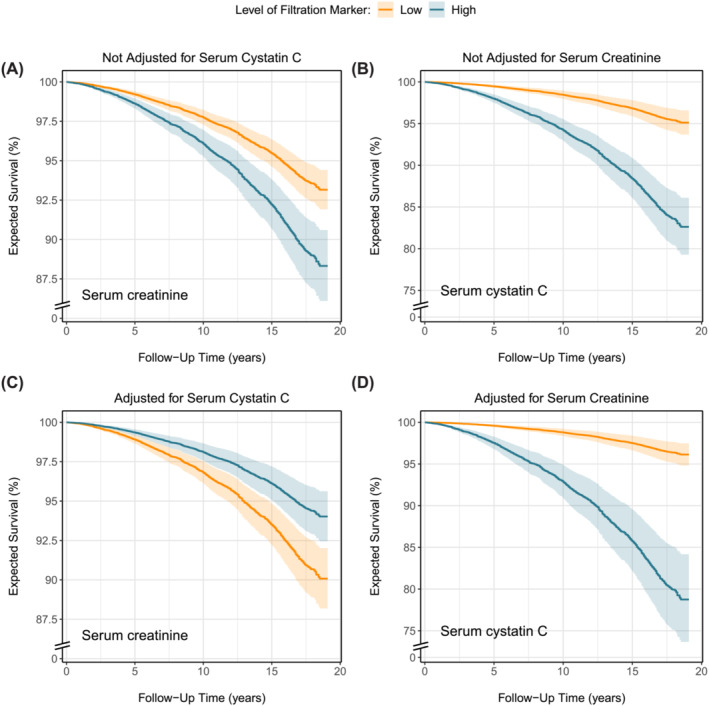
Effects of serum creatinine and cystatin C on the expected probability of survival with and without adjustment for each other in the replication cohort. Expected survival probabilities and associated 95% pointwise confidence intervals were derived from Cox models and were (aside from serum creatinine and/or cystatin C) adjusted for the baseline effects of age, sex, current smoking, alcohol consumption, history of malignancy, prevalent type 2 diabetes, history of cardiovascular disease, and waist circumference. Low and high levels of the filtration markers in the figure legend corresponded to the 2.5th and 97.5th percentile, which were 62 and 141 μmol/L for serum creatinine and 0.57 and 1.67 mg/L for serum cystatin C, respectively.

In the primary cohort, 1636 (19%) deaths were registered over a median (Q1–Q3) follow‐up of 12.9 (5.8–16.3) years with a 10‐year mortality rate (95% CI) of 7.6% (7.1–8.2%). Higher serum creatinine was minimally associated with decreased survival (Figure 3A), whereas higher serum cystatin C was strongly associated with decreased survival (Figure 3B); see also Table S2. When both markers were analysed together, higher serum creatinine was associated with increased survival (Figure 3C) and the association between higher serum cystatin C and decreased survival strengthened (Figure 3D). Hazard ratios of the associations are visualized in Figure S3. In the replication cohort, 1273 (25%) deaths were registered over a median follow‐up of 17.9 (17.3–18.5) years with a 10‐year mortality rate (95% CI) of 13.8% (12.8–14.7%). Higher serum creatinine as well as higher cystatin C were associated with decreased survival (Figure 4A,B). When both markers were adjusted for each other, higher serum creatinine was associated with increased survival (Figure 4C) and the association between higher serum cystatin C and decreased survival strengthened (Figure 4D). Hazard ratios of the associations are visualized in Figure S4.

## Discussion

Creatinine measures are the most commonly used proxy of kidney function.^22^, ^23^, ^24^ Circulating levels are related both to muscle mass and kidney function and must be separated from the former to fully reflect the latter. However, the inverse relationships between kidney function and muscle mass^5^, ^6^ may confound the accuracy of creatinine levels as marker of kidney function, if muscle mass deviates from typical values based on age and sex. In this study of two independent population‐based cohorts, higher serum creatinine was associated with greater muscle mass, whereas serum cystatin C was not associated with muscle mass. Notably, if both markers were analysed together, the positive association between serum creatinine and muscle mass strengthened and an inverse association between serum cystatin C and muscle mass became apparent. Likewise, both markers were positively associated with mortality, but when analysed together, higher serum creatinine was associated with decreased mortality, while the positive association between higher serum cystatin C and increased mortality strengthened.

The observation that low rather than high serum creatinine levels are associated with increased mortality has been previously described in dialysis patients.^13^, ^14^ In these patients, kidney function is essentially zero, meaning that serum creatinine levels purely reflect muscle mass (besides dietary ingestion).^12^ Here, we showed that serum creatinine strongly reflects muscle mass, even in circumstances of well‐preserved kidney function, challenging the use of creatinine measures as GFR marker.

**Figure 5:**
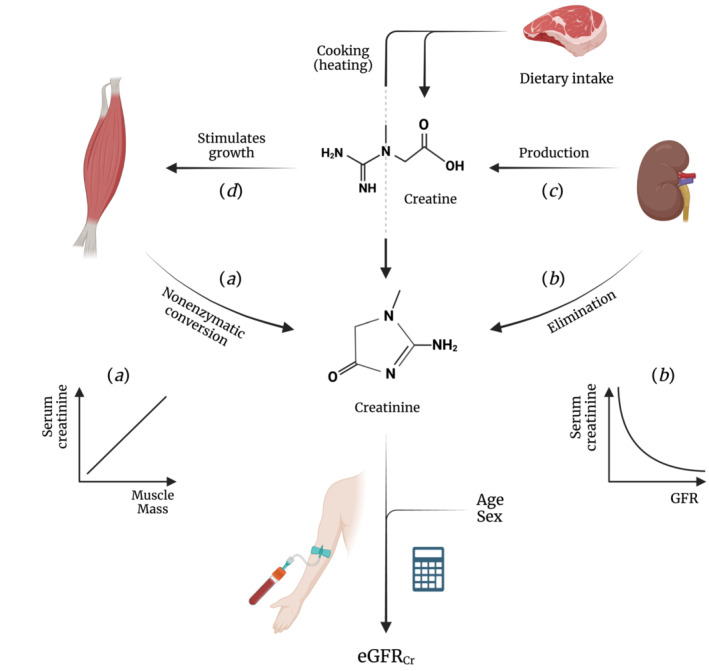
Schematic representation of the relationships between kidney function, muscle mass, and serum creatinine. This figure illustrates the complex pathways influencing circulating creatinine levels. Path *a* depicts the spontaneous, nonenzymatic conversion of creatine and phosphocreatine to creatinine in muscle, establishing a positive relationship between muscle mass and creatinine levels. Path *b* represents the elimination of creatinine an from the circulation by the kidneys, giving rise to the inverse relationship between GFR and circulating creatinine levels.^3^ Path *c* outlines the role of the kidney in creatine biosynthesis, beginning with the transfer of the amidino group from arginine to glycine to yield L‐ornithine and guanidinoacetate.^9^, ^10^ The amidino group of guanidinoacetate is then methylated in the liver to form creatine (not shown). Path *d* highlights the role of creatine in stimulating muscle protein synthesis. Both paths *c* and *d* reinforce the positive relationship between muscle mass and creatinine levels, highlighting the interconnectedness of kidney function, creatine production, and muscle mass. The calculator symbolizes GFR‐estimating equations that integrate age and sex with circulating creatinine levels to compute eGFRcr, thereby accounting for variability in creatinine related to muscle mass.^4^ eGFRcr, creatinine‐based eGFR. Created with BioRender.com

In steady‐state conditions, endogenous markers can be used to estimate GFR if circulating levels are inversely related to GFR and unrelated to other factors. Ideal markers should meet specific criteria: no tubular secretion or reabsorption, no extrarenal elimination, small molecular size, electric neutrality, no plasma protein binding, and constant production rate. Creatinine does not meet all criteria, given its tubular secretion, extrarenal elimination by gut microbiota, and variable production rate due to both dietary intake and the spontaneous, nonenzymatic conversion of both creatine and phosphocreatine in muscle tissue (Figure 5).^3^ Of these unmet criteria, the nonenzymatic conversion (Figure 5, path *a*) is the most problematic, as it makes circulating creatinine levels highly dependent on muscle mass. In our study, the positive associations of serum creatinine levels with muscle mass surrogates (Figures 1A,C and 2A,C) highlight the strong dependence on muscle mass. This dependence was further emphasized by reversal of the association between higher serum creatinine and increased mortality after cystatin C adjustment (Figures 3B and 4B). This adjustment largely detaches creatinine from its relationship with GFR, making the dependence on muscle mass stand out. Observing an inverse association between serum creatinine and mortality after cystatin C adjustment is not surprising, given that muscle mass is a reliable indicator of overall health^25^, ^26^ and frailty,^26^, ^27^ both strong prognosticators of mortality.^26^ A similar explanation underlies the positive associations between serum cystatin C and mortality (Figures 3C,D and 4C,D). Serum cystatin C is a robust marker of GFR.^3^ Given that impaired kidney function often coincides with co‐morbidities characterized by low muscle mass, a positive association between serum cystatin C and mortality is expected because of the increased prevalence of co‐morbidities and accompanying low muscle mass at higher cystatin C levels.^8^ This also explains the inverse associations of serum cystatin C with muscle mass surrogates (Figures 1D and 2D).

Two key processes fundamentally challenge the use of creatinine as a prime GFR marker. The first process involves the complexities of estimating GFR based on creatinine, especially when individual muscle mass deviates from standard expectations. Variability in serum creatinine across different individuals can be partially explained by age,^7^, ^28^, ^29^ sex,^7^, ^28^, ^29^, ^30^ and ethnic^28^, ^29^, ^30^, ^31^ differences in muscle mass.^32^ Estimating equations address some of the interindividual variability by including an individual's age and sex.^4^ However, these corrections are based on population‐level estimates and do not account for intra‐individual variations in muscle mass nor for situations where muscle mass deviates from typical values based on age and sex. Such circumstances can severely affect the accuracy of calculated eGFR as a proxy for true GFR.^7^ Notable examples include chronic illnesses (particularly CKD and conditions that contribute to its development^5^) and kidney donation, if kidney offers come from deceased donors of high age or with multimorbidity. Reliable evaluation of kidney function is crucial for detecting, evaluating, and monitoring acute kidney injury and CKD, selecting the correct dosage of drugs that are excreted by the kidney,^1^ and gaining insight in the expected GFR in kidney transplant recipients. Patients with chronic illnesses often require intensive GFR monitoring but also experience gradual muscle wasting,^8^ obviously undermining the reliability of eGFRcr. However, this undermining is not limited to situations of low muscle mass but extends to cases where muscle mass is higher than expected based on age and sex. For example, deceased young individuals, such as those who succumb to traffic accidents and could be potential kidney donors, may have higher‐than‐expected muscle mass. This could lead to the unjustified rejection of otherwise viable kidneys if GFR is approximated with eGFRcr.

Second, the interplay between circulating creatinine and kidney function goes beyond simple elimination. The biosynthesis of creatine, intimately linked to kidney function, adds another layer of complexity to using creatinine measures as GFR marker. Creatinine elimination gives rise to the well‐known, inverse relationship between serum creatinine and GFR (Figure 5, path *b*).^3^ However, it is much less known that kidney function is also *indirectly* related to circulating creatinine levels—namely, in a positive way. Kidney function is essential for the two‐step biosynthesis of creatine (Figure 5, path *c*). The first step primarily occurs in the kidney, where the amidino functionality of arginine is transferred to glycine to yield L‐ornithine and guanidinoacetate. The amidino functionality of guanidinoacetate is then methylated in the liver to form creatine (not shown).^9^, ^10^ The importance of the kidneys in creatine biosynthesis was initially indicated by a study, showing a 47% increase in guanidinoacetate levels in venous compared with arterial plasma from renal veins and arteries.^33^ Goldman and Moss subsequently demonstrated the rate‐limiting nature of this step by observing almost no creatine biosynthesis in nephrectomized animals.^34^

Besides its well‐known role as ATP reservoir, creatine also acts as crucial growth factor for stimulating muscle protein synthesis and maintaining muscle mass.^6^, ^9^, ^10^ This importance is strongly supported by observations in patients with rare genetic mutations that cause an inability to synthesize creatine, who have extraordinarily low muscle mass and strength and die from creatine depletion if not supplemented.^35^, ^36^ The rate of creatine (and hence creatinine) generation therefore heavily depends on kidney function, depicted in Figure 5 by paths *c* through *a*. It is this positive relationship between kidney function and muscle mass that further challenges the reliability of creatinine measures as marker of GFR, as its generation and elimination are both directly linked to GFR.

In settings where eGFRcr is considered less accurate, confirmatory testing with cystatin C is recommended.^1^ Our findings show that cystatin C is hardly (Figures 1B and 2B) associated with muscle mass when not adjusted for serum creatinine. This supports its utility in such contexts and aligns with recent advocacy from two US national kidney disease organizations for more routine use of cystatin C.^37^ However, increased use of cystatin C‐based eGFR (eGFRcys) can result in eGFR values that differ substantially from those derived from creatinine. In such cases, it becomes challenging for physicians to determine which value is more trustworthy. Discordant values can arise from various sources, such as rounding of or measurement errors in creatinine or cystatin C levels, assumptions underlying GFR‐estimating equations, and (unidentified) factors accounting for variability in creatinine and cystatin C unrelated to GFR, referred to as ‘non‐GFR determinants’.^38^ A recent study that leveraged real‐world data suggested that the estimating equation that combines both markers (eGFRcr‐cys) is more accurate than either eGFRcr or eGFRcys in cases of high discordance.^39^ A major limitation of the study was the lack of data on why patients were referred for assessment of creatinine, cystatin C, and measured GFR, which both hampered determining the more accurate eGFR and appraisal of the extent to which non‐GFR determinants may have confounded results. While we support the use of cystatin C for confirmatory testing when eGFRcr is less accurate, we reserve judgement on the preference between eGFRcys and eGFRcr‐cys. Further research should provide definitive answers.

Our study has some limitations. First, 24‐hour urine specimens might have been collected with error. Nonetheless, participants received thorough instruction prior to each screening round and the use of paired 24‐hour urine specimens, separated by 3 weeks, should have brought potential measurement error to a minimum. Second, DXA‐derived appendicular lean soft tissue may be biased by abnormal hydration status. However, the similarity in the associations of kidney function makers with CER index and DXA‐derived appendicular lean soft tissue suggests that the impacts of the foregoing limitations, if any, are minimal. Third, the observational nature of our study hampers inferring causality or ruling out residual confounding in the observed associations of serum creatinine and cystatin C with CER index and DXA‐derived appendicular lean soft tissue and with mortality. Fourth, no data on GFR measured with exogenous filtration markers was available, which hampered adjusting the associations for golden standard surrogates of kidney function.

In conclusion, our study demonstrates a strong association between higher serum creatinine levels and greater muscle mass, challenging the reliability of creatinine measures as GFR marker and suggesting the need for a more cautious approach in its clinical use. Conversely, serum cystatin C demonstrates minimal association with muscle mass, supporting its increased use in routine clinical practice as a more reliable alternative. Future research focused on jointly quantifying non‐GFR determinants of creatinine and cystatin C could offer valuable insights, paving the way for more personalized and accurate GFR assessments.

## Funding

None.

## Conflict of interest

The authors declare that they have no conflict of interest.

## Supporting information


**Box S1.** Details on procedure of multiple imputation of missing observations in covariates.
**Figure S1.** Flow of participants through the PREVEND study.
**Figure S2.** Flow of participants through the NHANES.
**Figure S3.** Graphical representation of the association of serum creatinine and cystatin C with CER index
**Figure S4.** Graphical representation of the associations of serum creatinine and cystatin C with all‐cause mortality in the primary cohort.
**Figure S5.** Graphical representation of the associations of serum creatinine and cystatin C with all‐cause mortality in the replication cohort.
**Table S1.** Effect estimates from least‐squares regression models for surrogates of muscle mass*.
**Table S2.** Effect estimates from Cox proportional hazards models for all‐cause mortality*.
