# Supplementary material for: Creatinine, cystatin C, muscle mass, and mortality: Findings from a primary and replication population‐based cohort
Source: J Cachexia Sarcopenia Muscle. 2024 Jun 19;15(4):1528–38. doi: 10.1002/jcsm.13511 (PMC11294032; doi:10.1002/jcsm.13511)
Supplement: Supplementary file 1 — Box S1. Details on procedure of multiple imputation of missing observations in covariates. Figure S1. Flow of participants through the PREVEND study. Figure S2. Flow of participants through the NHANES. Figure S3. Graphical representation of the association of serum creatinine and cystatin C with CER index Figure S4. Graphical representation of the associations of serum creatinine and cystatin C with all‐cause mortality in the primary cohort. Figure S5. Graphical representation of the associations of serum creatinine and cystatin C with all‐cause mortality in the replication cohort. Table S1. Effect estimates from least‐squares regression models for surrogates of muscle mass*. Table S2. Effect estimates from Cox proportional hazards models for all‐cause mortality*. [file JCSM-15-1528-s001.docx]

**Table of Contents**

**File S1.** Study Methods

Study cohorts and data collection ……………….………………………………………………………. 3

Statistical Analyses ……………………………………………………………………………………….. 6

**Figure S1.** Flow of participants through the PREVEND study ……………………………………………….. 10

**Figure S2.** Flow of participants through the NHANES ………………………………………………………… 11

**Figure S3.** Graphical representation of the association of serum creatinine and cystatin C with CER index …. 12

**Figure S4.** Graphical representation of the association of serum creatinine and cystatin C with mortality in the primary cohort. …………………………………………………………………………………………………... 13

**Figure S5.** Graphical representation of the association of serum creatinine and cystatin C with mortality in the replication cohort. ……………………………………………………………………………………………….. 14

**Table S1.** Effect estimates from least-squares regression models for surrogates of muscle mass .…………………………………..………………………………………………………………………………. 15

**Table S2.** Effect estimates from Cox proportional hazards models for all-cause mortality …………………... 16

**References** …………………………………………………………………………………………………….. 17

**File S1. Study Methods**

Groothof D, Shehab NBN, Erler NS, Post A, Kremer D, Polinder-Bos HA *et al*. Creatinine, cystatin C, muscle mass, and mortality: findings from a primary and replication population-based cohort

These supplemental methods have been provided by the authors to give readers additional information about their work.

**Study cohorts and data collection**

**The Prevention of REnal and Vascular ENd-stage Disease (PREVEND) study**

*Design*

The PREVEND study prospectively investigates risk factors for and the prevalence and consequences of microalbuminuria in otherwise healthy adults in the city of Groningen (the Netherlands). In 1997-1998, all 85,421 inhabitants of the city of Groningen aged 28-75 years were invited to participate in the study and were asked to complete a brief questionnaire and provide morning urine. The urinary albumin concentration (UAC) was determined in 40,856 responders, 9,966 of whom had a UAC greater than or equal to 10 mg/l and 30,890 had a UAC less than 10 mg/l. Pregnant women and participants with insulin-dependent diabetes mellitus were excluded (because these conditions are known to influence the UAC). The resulting 7,768 participants with a UAC greater than or equal to 10 mg/l were requested to participate in the cohort, of whom 6,000 were enrolled. Additionally, 3,395 of the 22,350 eligible participants with a UAE less than 10 mg/l were randomly chosen and invited to serve as a control group was invited, of whom 2,592 were enrolled. The size of this random sample was arbitrarily set at 3,395 participants to obtain a total cohort size of approximately 10,000 participants, taking into account a 15% nonparticipation rate. These 8,592 participants attended the first screening of the PREVEND study. This study included 8,437 participants with available data on urinary creatinine measurements, racial status, and follow-up data on mortality (**Figure S1**).

*Data collection and definitions*

The study consisted of five consecutive screening rounds, each screening comprising two visits to an outpatient clinic separated by three weeks. Self-administered questionnaires concerning demographics, cardiovascular and kidney disease history, smoking habits, and medication use were provided by all participants prior to the first visit. Information on medication use was combined with information from IADB.nl, a pharmacy-dispensing registry containing information of prescribed medication in public pharmacies in the Netherlands since 1994 (http://www.iadb.nl/). Educational level was categorized into low (no, primary, basic vocational and secondary education), middle (senior secondary vocational and general senior secondary education), and high (higher professional and higher academic education) according to the International Standard Classification of Education [1]. Alcohol consumption was defined as consuming 10 grams of ethanol or more per day. Systolic and diastolic blood pressure were measured on the right arm, every minute during 10 minutes in the first visit and 8 minutes in the second visit, using an automatic Dinamap XL Model 9300 series device (Johnson-Johnson Medical Inc., Tampa, FL, USA) and calculated as the mean of the last two measurements of the two visits. Weight was measured to the nearest 0.5 kg with a Seca balance scale (Vogel and Halke, Hamburg, Germany) after shoes and heavy clothing had been taken off. Height was measured to the nearest 0.5 cm. Minimal waist circumference was measured on bare skin at the natural indentation between the 10^th^ rib and the iliac crest. Type 2 diabetes was defined as a fasting plasma glucose greater than 7.0 mmol/l, non-fasting plasma glucose greater than 11.1 mmol/l, self-report of a physician’s diagnosis, or the use of antidiabetic drugs. Fasting was defined as not having eaten or drunk anything other than plain water for eight hours or more. Prevalent cardiovascular disease comprised the events of myocardial infarction, ischemic heart disease, coronary artery bypass grafting, percutaneous coronary intervention, subarachnoid hemorrhage, intracerebral hemorrhage, occlusion and stenosis of precerebral and cerebral arteries, carotid endarterecotomy, aorta peripheral bypass surgery, and percutaneous transluminal femoral angioplasty.

Participants had to collect two consecutive 24-hour urine specimens after thorough oral and written instruction. During collection, participants were asked to refrain from heavy exercise and instructed to postpone urine collection in case of urinary tract infection, menstruation, or fever. Collected urine was subsequently stored cold (4 °C) for a maximum of four days before the second visit. Specimens of the urine collections were stockpiled at -20 °C until analysis. Blood samples were drawn between 8:00 and 10:00 a.m. from all participants and aliquots were immediately stockpiled at -80 °C until analysis. Serum creatinine was measured with an enzymatic method on a Roche Modular analyzer, using reagents and calibrators from Roche (Roche Diagnostics, Mannheim, Germany). Coefficients of variation (CVs) for total imprecision at 85 μmol/l and 394 μmol/l were 1.2% and 1.1%, respectively. Serum cystatin C was measured with the Gentian Cystatin C Immunoassay (Gentian AS, Moss, Norway) on a Roche Modular analyzer and was calibrated directly with the standard supplied by the manufacturer. The CVs for total imprecision at 0.85 and 2.97 mg/l were 1.8% and 3.0%, respectively. Urinary creatinine was measured by dry chemistry (Eastman Kodak, Rochester, USA) with a CV of total imprecision of 2.9%. Urinary albumin was measured by immunonephelometry (Dade Behring BNII, Marburg, Germany) with a lower limit of detection of 2.3 mg/l and CV for total imprecision of 4.4%. The urinary albumin and creatinine concentrations were multiplied by urine volume to obtain values in mg and mmol per 24 hours, respectively. These values are here referred to as ‘urinary albumin excretion’ and the ‘creatinine excretion rate’ (CER). The mean value of the paired 24-hour urine collections was calculated for each screening round. The CER was subsequently used to approximate total-body skeletal muscle mass using the equation 18.9 $\times$ CER + 4.1, with CER expressed in grams of urinary creatinine excreted in 24 hours [2]. As a second surrogate of muscle mass, the CER was indexed by height as previously described [3]. This height-indexed CER is hereafter referred to as ‘CER index’.

**The National Health and Nutrition Examination Survey (NHANES)**

*Design*

The NHANES comprises a biannual survey designed to evaluate the health of children and adults in a representative sample of the noninstitutionalized civilian U.S. population [4]. Details on the design of the sample can be found elsewhere [5]. Data collected through in-home interviews and study visits at mobile examination centers have been released in 2-year cycles. This study used data from the 2001-2002 cycle of the continuous NHANES. Adult participants aged 18 years or older with available data on education, serum creatinine, serum cystatin C, dual energy X-ray absorptiometry (DXA), and follow-up data on mortality were included (**Figure S2**). Detailed descriptions of NHANES methods and data access are publicly available on the NHANES website [6]. The National Health Statistics Research Ethics Review Board approved NHANES (Protocol #98-12) [7]. Written informed consent was obtained from all adult participants.

*Data collection and definitions*

Information on age, sex, race, education, and medical conditions was collected during a household interview. Educational level was categorized into less than high school, high school diploma (including GED), and more than high school. Alcohol consumption was defined as having had 12 or more drinks in any one year. Smoking was defined as having smoked at least 100 cigarettes in life. Type 2 diabetes was defined as a fasting plasma glucose greater than 7.0 mmol/l, non-fasting plasma glucose greater than 11.1 mmol/l, self-report of a diabetes diagnosis by a physician or other health professional, or the use of insulin. Fasting was defined as not having eaten or drunk anything other than plain water for eight hours or more. Prevalent cardiovascular disease was defined as having a history of congestive heart failure, coronary artery disease, angina/angina pectoris, heart attack, and/or stroke. History of malignancy was defined as self-report of a cancer diagnosis by a physician or other health professional. Height, weight, waist circumference, and blood pressure, were measured during a subsequent visit to a mobile examination center according to standardized protocols [8,9]. Weight was measured to the nearest 100 g with a digital weight scale (or two portable scales if weight exceeded 200 kg) after shoes had been taken off and the standard mobile examination center examination gown (disposable shirt, pants, and slippers) had been put on. Height was measured in straight standing position to the nearest 0.1 cm. Minimal waist circumference was measured on bare skin just above the uppermost lateral border of the right ilium to the nearest 0.1 cm. Systolic and diastolic blood pressure were measured on the right arm unless specific known or self-reported conditions prohibited the use of the right arm. Three consecutive blood pressure readings (or four in case of interruption or inability of getting one or more readings) were obtained using the same arm, with at least 30 seconds in between readings. Whole body DXA scans were taken with a Hologic QDR-4500A fan-beam densitometer (Hologic, Inc., Bedford, Massachusetts). Hologic software version 8.26:a3* was used to administer all scans. The densitometer scanned participants with an X-ray source using fan-beam scan geometry in three passes (1 minute per pass). The participants were positioned supine on the tabletop with their feed in a neutral position and hands flat by their side. A Velcro strap was used to keep the feet stationary and together. The DXA technique acquires two low-dose X-ray images at different average energies. The ratio of the attenuation of these two average energies, called an R-factor, is used to distinguish both bone from soft tissue, and the percent fat in soft tissue when bone is not present. The radiation exposure from DXA is extremely low at less than 10 µSv. The sum of the upper and lower extremity DXA-derived appendicular lean soft tissue (ALST) were used to approximate total-body skeletal muscle mass expressed in kg using the equation 1.19 $\times$ ALST – 1.01 [10].

Blood was obtained by a trained phlebotomist according to a standardized protocol [11]. Serum creatinine was measured with a Jaffe kinetic alkaline picrate method performed on a Beckman LX-20 analyzer and a Roche Hitachi 917 analyzer. The CVs for intra-assay and inter-assay imprecision were 2.2-3.1% and 3.5-4.6%, respectively. This method, although not standardized across different laboratories, has been shown to be consistent within acceptable limits. A comparative study using serum creatinine assay from NHANES 2001-2002, which utilized the Jaffe kinetic rate method, and a Roche coupled enzymatic assay method performed on a Roche P Module instrument and traceable to a gold standard reference, revealed no significant differences in results. The regression analysis between the two methods showed an intercept and slope within the analytical error margin of the NHANES method (CVs of 4.4% and 2.9% at 0.68 mg/dL and 1.26 mg/dL, respectively). Consequently, no standardization or correction was deemed necessary for serum creatinine in this context. Serum cystatin C was measured with the Dade Behring N Latex Cystatin C assay, which is an automated particle-enhanced nephelometric assay run on the Dade Behring Nephelometer II [12]. The CVs for intra-assay and inter-assay imprecision were 2.0-3.0% and 3.2-4.4%, respectively. The assay range was 0.23-7.25 mg/l.

**Statistical Analyses**

*Multiple imputation*

To account for and reduce potential bias due to missing data [13], multiple imputation of incomplete covariates using Substantive Model Compatible Fully Conditional Specification was performed with the R package ‘smcfcs’ [14], to obtain 10 imputed data sets for data from the PREVEND study. For the NHANES data, the situation was more complex due to the unique characteristics of the dual energy X-ray absorptiometry (DXA) data.
Specifically, missing DXA data had already previously been multiply imputed five times because of relatively large amounts of missing DXA data along with systemic, nonrandom patters to the missing data [15]. Here, we merged these five sets with other NHANES data, ensuring that the only variation between them was in the DXA values. We then multiply imputed each of the resulting five merged data sets 10 times, culminating in a total of 50 complete data sets. This larger number of multiply imputed data sets accounts for the large proportion of missing observations in serum cystatin C, as detailed in **Table 1** of the main manuscript. For both PREVEND and NHANES data, the algorithm was run 30 iterations. All continuous variables were standardized to facilitate convergence and were back-transformed afterwards. Convergence of the Markov chains was evaluated with trace plots of the substantive model parameters. To confirm that imputed values were biologically plausible, the distributions of the imputed values were visually investigated and compared to the distribution of the observed values. Analyses were performed in each of the data sets and results were pooled using Rubin’s rules [16,17]. Detailed information on the imputation procedure is given below in **Box S1**.

| **Box S1. Details on procedure of multiple imputation of missing observations in covariates** | | | | |
| --- | --- | --- | --- | --- |
|  |  | **Cohort** |  |  |
| Software used |  |  |  | R version 4.3.3 |
| Imputation method and key settings |  |  |  | Substantive Model Compatible Fully Conditional Specification (package: ‘smcfcs’ version 1.7.1) [14]; iterations: 30 |
| Number of imputed data sets |  | PREVEND |  | 10 |
|  |  | NHANES |  | 50 |
| Substantive model |  |  |  | Cox proportional hazards model |
| Analysis variables |  | PREVEND |  | Age; alcohol consumption; all-cause mortality; current smoking; follow-up time; history of cardiovascular disease; prevalent malignancy; prevalent type 2 diabetes; race; log_2_ serum creatinine; log_2_ serum cystatin C; sex; log_2_ urinary albumin excretion; waist circumference |
|  |  | NHANES |  | Age; alcohol consumption; all-cause mortality; follow-up time; history of malignancy; prevalent cardiovascular disease; ln serum creatinine; ln serum cystatin C; sex; smoked at least 100 cigarettes in life; waist circumference |
| Auxiliary variables |  | PREVEND |  | Education; diastolic blood pressure; height; high-density lipoprotein cholesterol; systolic blood pressure; total cholesterol; ln triglycerides; weight; use of antihypertensive drugs; use of lipid-lowering drugs; 24-hour urinary creatinine; 24-hour urinary volume; |
|  |  | NHANES |  | Education; diastolic blood pressure; high-density lipoprotein cholesterol; systolic blood pressure; total cholesterol; ln triglycerides; total-body skeletal muscle mass; weight |
| Treatment of continuous variables |  |  |  | Linear regression |
| Treatment of binary variables |  |  |  | Logistic regression |
| Population |  | PREVEND |  | Participants who attended the first screening and had available data on urinary creatinine excretion, race, and follow-up data on mortality (n=8,437) |
|  |  | NHANES |  | Participants from the 2001-2002 NHANES cycle with available data on education, DXA, and follow-up data on mortality (n=5,033) |

DXA, dual energy X-ray absorptiometry.

*Linear regressions*

Baseline effects of log­_2_ serum creatinine and cystatin C on muscle mass surrogates were quantified using linear regression models, specifying sex, current smoking, alcohol consumption, prevalent malignancy, prevalent type 2 diabetes mellitus, history of cardiovascular disease, serum creatinine, serum cystatin C, age, waist circumference, and log­_2_ urinary albumin excretion as main effects as well as a product term between age and sex. Natural cubic splines with two degrees of freedom were used to model potential nonlinear effects of log_2_ serum creatinine, log_2_ serum cystatin C, age, waist circumference, and log_2_ urinary albumin excretion. Boundary knots were set to the 2.5^th^ and 97.5^th^ percentile of the relevant variables. Model simplification was done using multivariate Wald tests. The resulting point estimates, confidence intervals, and *P* values are presented in **Table S1**. Moreover, results are visualized to facilitate their interpretation. Model assumptions were validated by plotting residuals against the fitted values and every covariate. Normality of residuals was evaluated by inspection of Q-Q plots.

*Cox proportional hazards regressions*

Effects of log_2_ serum creatinine and cystatin C on the baseline hazard of death were quantified with Cox proportional hazards models, specifying sex, current smoking, alcohol consumption, prevalent malignancy, prevalent type 2 diabetes, and nonlinear effects of serum creatinine, serum cystatin C, age, waist circumference, and urinary albumin excretion as main effects. Two potential interactions were explored by introducing product terms of age with sex and waist circumference. Natural cubic splines with two degrees of freedom were used to model potential nonlinear effects of log_2_ serum creatinine, log_2_ serum cystatin C, age, waist circumference, and log_2_ urinary albumin excretion. Boundary knots were set to the 2.5^th^ and 97.5^th^ percentile of the relevant variables. Point estimates, confidence intervals, and *P* values are presented in **Table S2**. The results are visualized to facilitate their interpretation. The proportional hazards assumption was validated through inspection of Schoenfeld residuals plots, revealing that the assumption was met for every covariate.


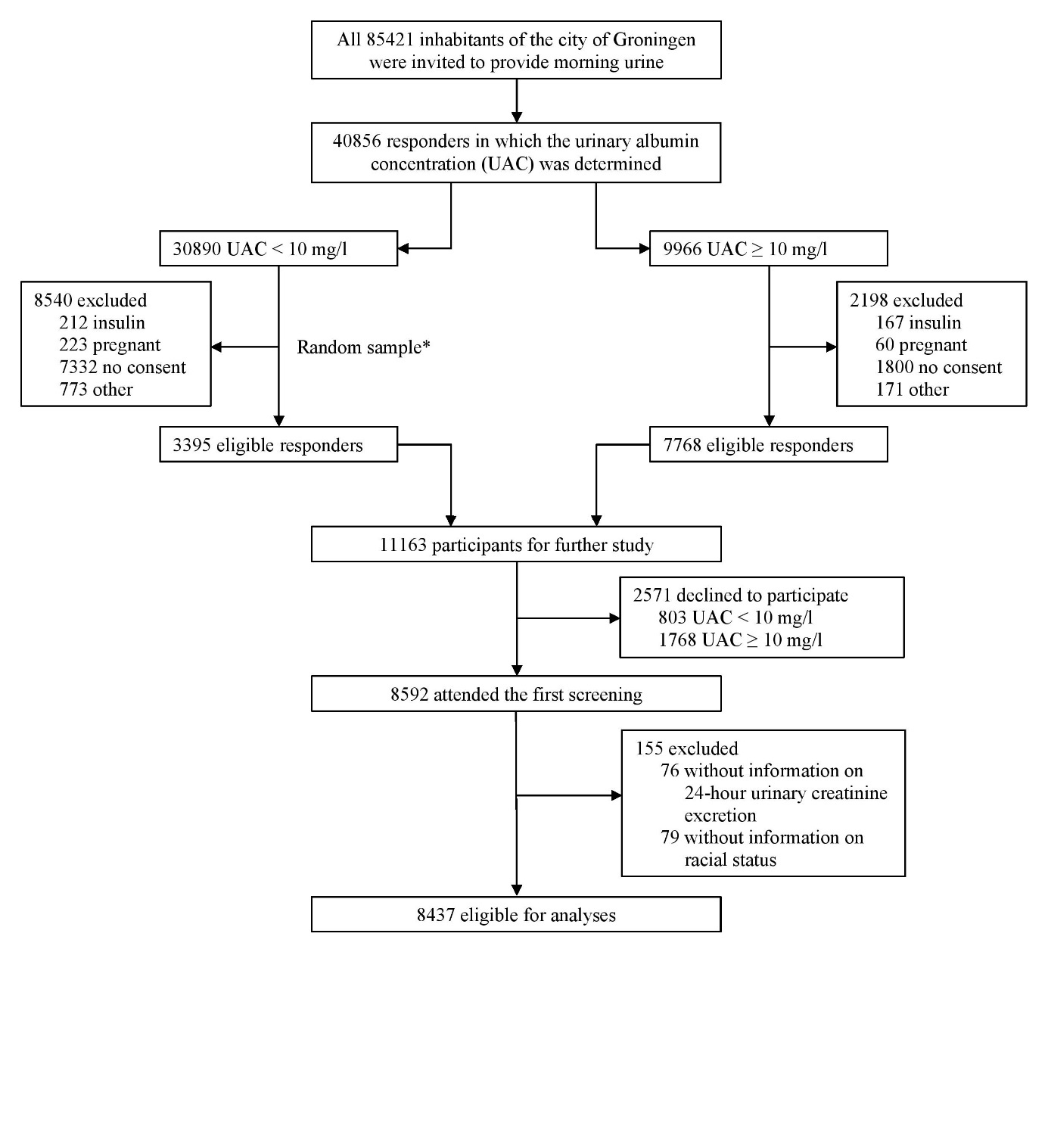


**Figure S1. Flow of participants through the PREVEND study**

*Size of the random sample was arbitrarily set at 3,395 (out of the 22,350 eligible participants) to obtain a total cohort size of approximately 10,000, taking into account a 15% nonparticipation rate.


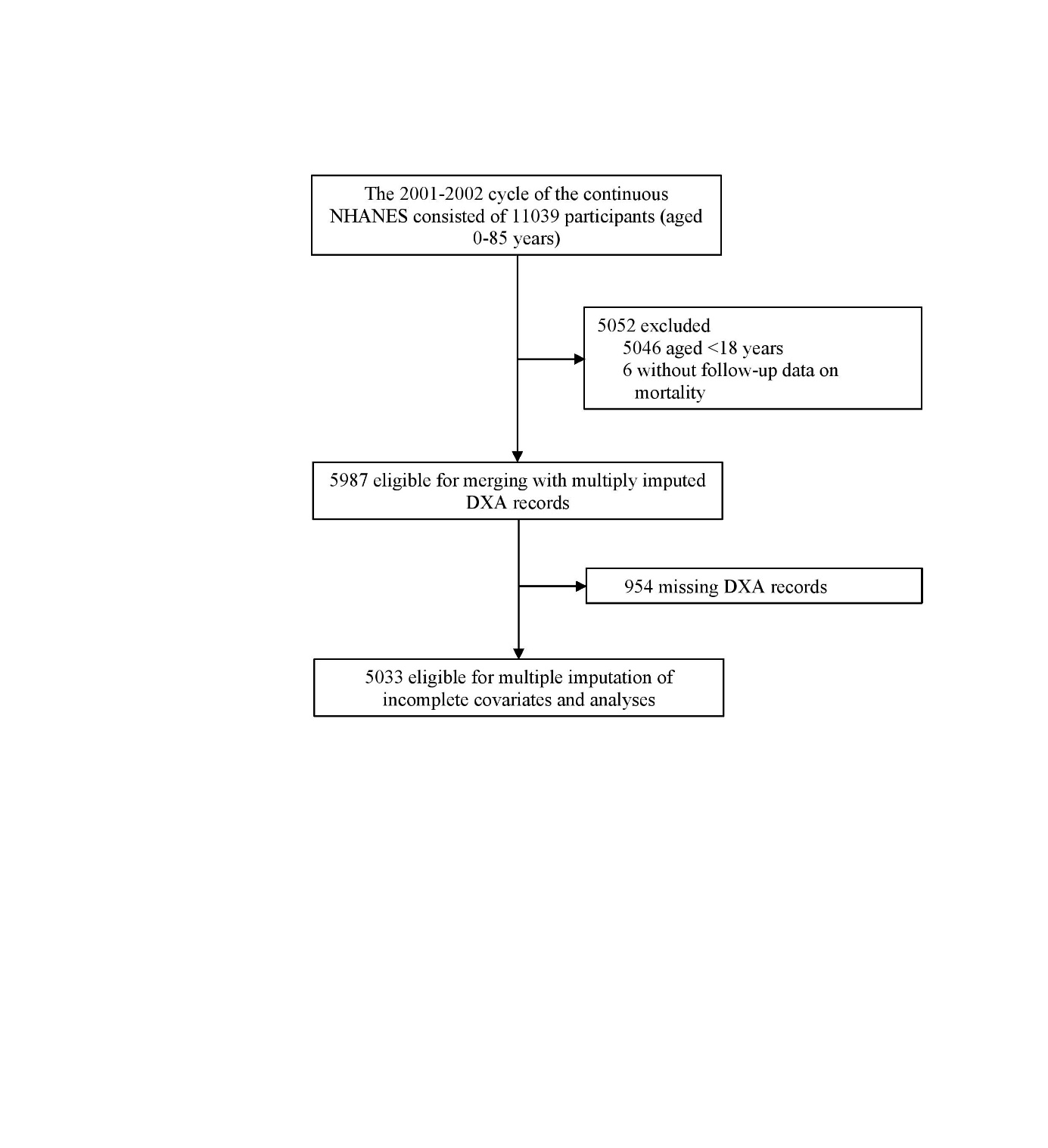


**Figure S2. Flow of participants through the NHANES**

DXA, dual energy X-ray absorptiometry.


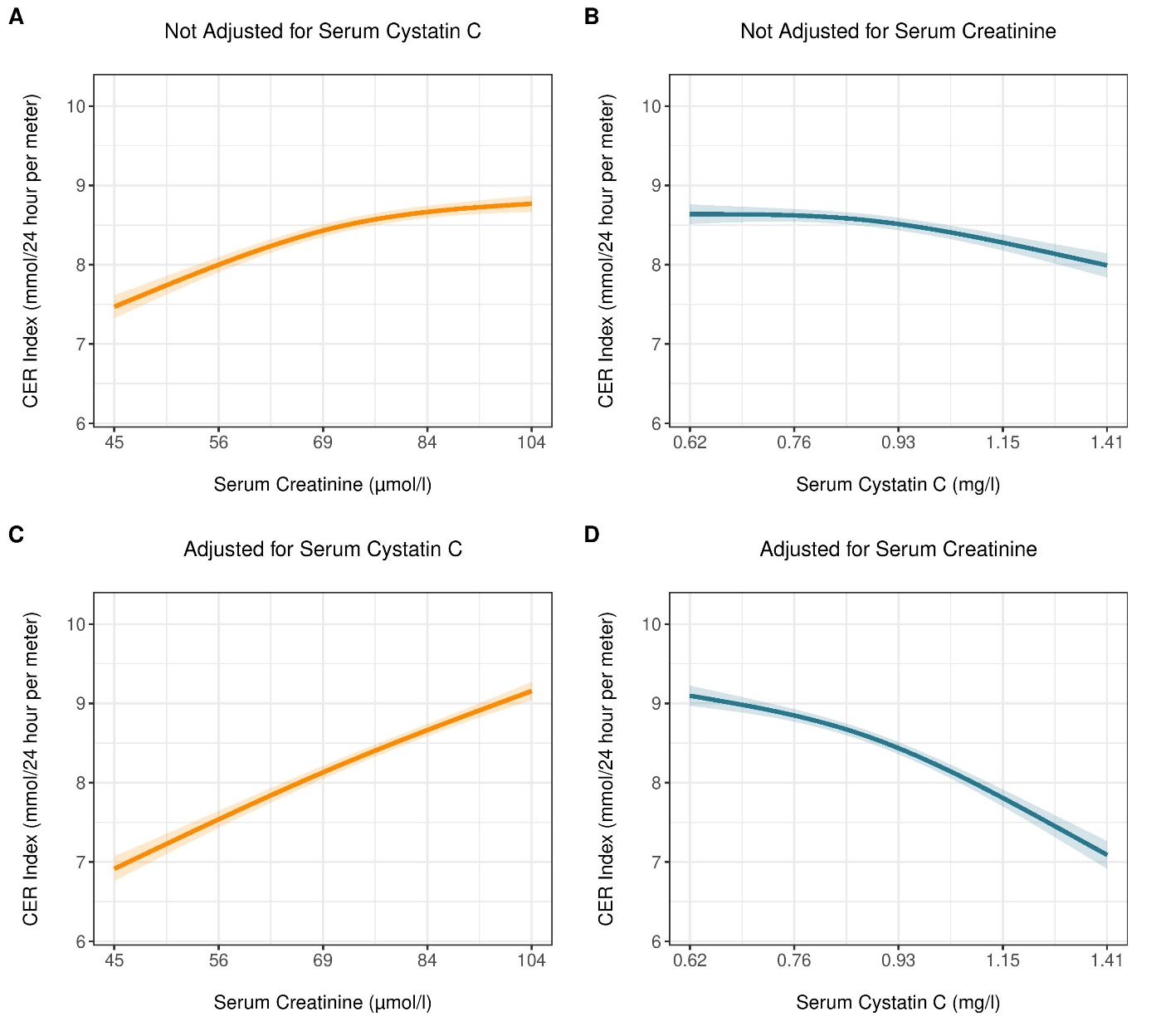


**Figure S3 Graphical representation of the association of serum creatinine and cystatin C with CER index**

Expected CER indices and associated 95% pointwise confidence intervals were derived from linear regression models and were (aside from serum creatinine and/or cystatin C) adjusted for the effects of age, sex, current smoking, alcohol consumption, prevalent malignancy, prevalent type 2 diabetes, history of cardiovascular disease, waist circumference, and urinary albumin excretion. CER index, height-indexed creatinine excretion rate.


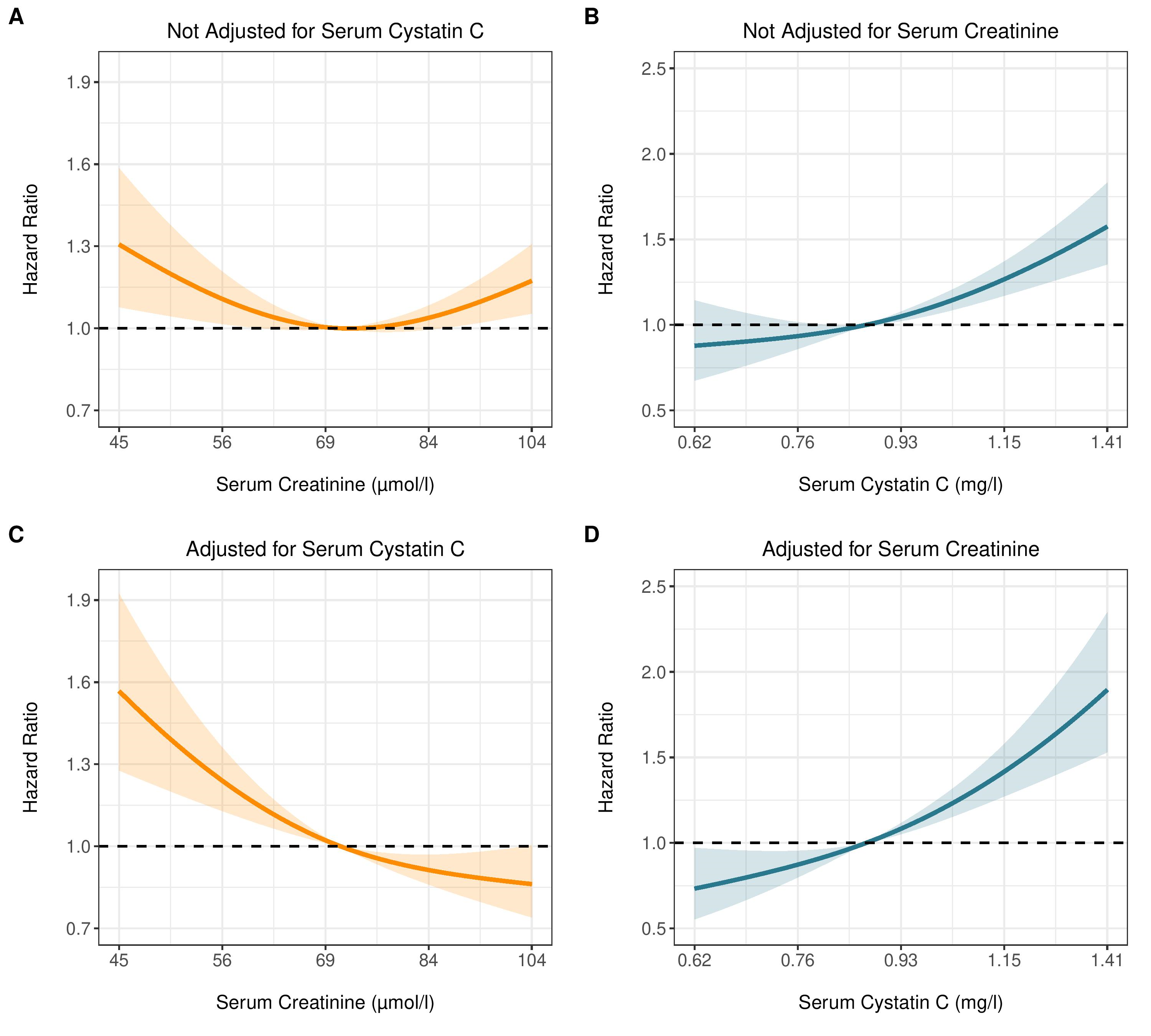


**Figure S4. Graphical representation of the associations of serum creatinine and cystatin C with all-cause mortality in the primary cohort**. Hazard ratios and associated 95% pointwise confidence intervals were derived from Cox models and were (aside from serum creatinine and/or cystatin C) adjusted for the baseline effects of age, sex, current smoking, alcohol consumption, prevalent malignancy, prevalent type 2 diabetes, history of cardiovascular disease, waist circumference, and urinary albumin excretion. The median level of the relevant kidney function marker was chosen as a reference.


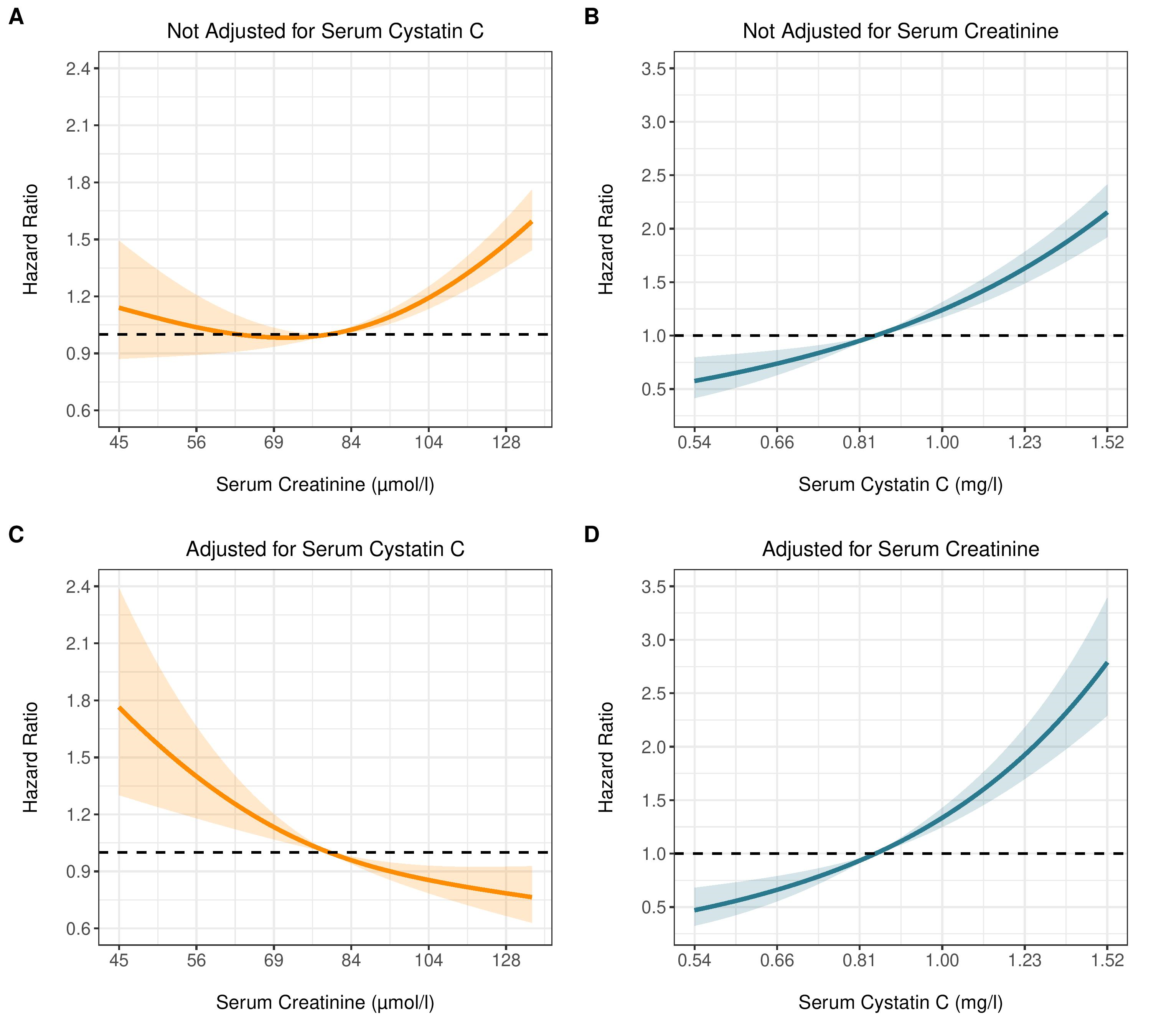


**Figure S5. Graphical representation of the associations of serum creatinine and cystatin C with all-cause mortality in the replication cohort**. Hazard ratios and associated 95% pointwise confidence intervals were derived from Cox models and were (aside from serum creatinine and/or cystatin C) adjusted for the baseline effects of age, sex, current smoking, alcohol consumption, history of malignancy, prevalent type 2 diabetes, history of cardiovascular disease, waist circumference. The median level of the relevant kidney function marker was chosen as a reference.

| **Table S1.** **Effect estimates from least-squares regression models for surrogates of muscle mass*** | | | | | | | | |
| --- | --- | --- | --- | --- | --- | --- | --- | --- |
|  | | | | | | | | |
|  |  | | **PREVEND** | | | | **NHANES** | |
|  |  | **Log_2_ skeletal muscle mass** | | | **Height-indexed CER** | | **Log_2_ skeletal muscle mass** | |
|  |  | **b (95% CI)** | | ***P* value** | **b (95% CI)** | ***P* value** | **b (95% CI)** | ***P* value** |
|  |  |  | |  |  |  |  |  |
| Intercept |  | 3.90 (3.86 to 3.95) | | <0.001 | 1.91 (1.67 to 2.15) | <0.001 | 3.45 (3.40 to 3.50) | <0.001 |
| N(serum creatinine)_1_ |  | 0.55 (0.51 to 0.59) | | <0.001 | 2.68 (2.45 to 2.90) | <0.001 | 0.65 (0.60 to 0.71) | <0.001 |
| N(serum creatinine)_2_ |  | 0.28 (0.26 to 0.31) | | <0.001 | 1.59 (1.45 to 1.73) | <0.001 | 0.32 (0.29 to 0.36) | <0.001 |
| N(serum cystatin C)_1_ |  | -0.25 (-0.30 to -0.21) | | <0.001 | -1.48 (-1.72 to -1.25) | <0.001 | -0.22 (-0.29 to -0.16) | <0.001 |
| N(serum cystatin C)_2_ |  | -0.27 (-0.30 to -0.24) | | <0.001 | -1.54 (-1.69 to -1.38) | <0.001 | -0.23 (-0.27 to -0.19) | <0.001 |
| N(age)_1_ |  | -0.35 (-0.40 to -0.31) | | <0.001 | -1.49 (-1.73 to -1.26) | <0.001 | -0.36 (-0.39 to -0.33) | <0.001 |
| N(age)_2_ |  | -0.33 (-0.36 to -0.30) | | <0.001 | -1.35 (-1.51 to -1.19) | <0.001 | -0.42 (-0.45 to -0.40) | <0.001 |
| Male sex |  | 0.21 (0.19 to 0.24) | | <0.001 | 0.83 (0.68 to 0.98) | <0.001 | -0.38 (-0.39 to -0.36) | <0.001 |
| Smoking behavior |  | -0.01 (-0.02 to 0.00) | | 0.10 | 0.01 (-0.06 to 0.07) | 0.84 | 0.02 (0.00 to 0.03) | 0.016 |
| Alcohol consumption |  | -0.01 (-0.02 to 0.01) | | 0.37 | -0.08 (-0.14 to -0.01) | 0.020 | 0.01 (-0.01 to 0.02) | 0.28 |
| Waist circumference |  | 0.01 (0.01 to 0.01) | | <0.001 | 0.04 (0.04 to 0.05) | <0.001 | 0.01 (0.01 to 0.01) | <0.001 |
| Prevalent type 2 diabetes |  | 0.00 (-0.03 to 0.03) | | 0.87 | 0.05 (-0.10 to 0.20) | 0.50 | -0.02 (-0.04 to 0.00) | 0.030 |
| Prevalent cardiovascular disease |  | -0.06 (-0.08 to -0.04) | | <0.001 | -0.32 (-0.45 to -0.19) | <0.001 | 0.01 (-0.01 to 0.03) | 0.47 |
| Prevalent cancer |  | -0.05 (-0.09 to 0.00) | | 0.029 | -0.27 (-0.49 to -0.04) | 0.019 | -0.01 (-0.03 to 0.01) | 0.20 |
| N(urinary albumin excretion)_1_ |  | 0.43 (0.40 to 0.47) | | <0.001 | 2.19 (2.01 to 2.36) | <0.001 | - | - |
| N(urinary albumin excretion)_2_ |  | 0.04 (0.02 to 0.06) | | <0.001 | 0.24 (0.13 to 0.35) | <0.001 | - | - |
| N(age)_1_ × sex |  | 0.13 (0.07 to 0.19) | | <0.001 | 0.63 (0.31 to 0.96) | <0.001 | - | - |
| N(age)_2_ × sex |  | 0.06 (0.03 to 0.10) | | <0.001 | -0.03 (-0.22 to 0.17) | 0.78 | - | - |
|  | | | | | | | | |
| *A dash (-) indicates that the specific model term not included in the final model. N(·)_1_ and N(·)_2_ denote the basis for a natural cubic spline with two degrees of freedom and natural boundary conditions imposed on the 2.5^th^ and 97.5^th^ percentiles of the relevant variable to model potential nonlinearities. CER, 24-hour creatinine excretion rate. | | | | | | | | |

| **Table S2. Effect estimates from Cox proportional hazards models for all-cause mortality*** | | | | | |
| --- | --- | --- | --- | --- | --- |
|  | | | | | |
|  |  | **Primary cohort** | | **Replication cohort** | |
|  |  | **Log HR (95% CI)** | ***P* value** | **Log HR (95% CI)** | ***P* value** |
|  |  |  |  |  |  |
| N(serum creatinine)_1_ |  | -0.80 (-1.17 to -0.42) | <0.001 | -1.14 (-1.77 to -0.52) | <0.001 |
| N(serum creatinine)_2_ |  | -0.34 (-0.55 to -0.13) | 0.001 | -0.52 (-0.79 to -0.25) | <0.001 |
| N(serum cystatin C)_1_ |  | 0.83 (0.27 to 1.39) | 0.004 | 2.09 (1.04 to 3.14) | <0.001 |
| N(serum cystatin C)_2_ |  | 0.71 (0.48 to 0.94) | <0.001 | 1.53 (1.15 to 1.91) | <0.001 |
| N(age)_1_ |  | 4.57 (2.74 to 6.41) | <0.001 | 5.89 (4.23 to 7.55) | <0.001 |
| N(age)_2_ |  | 3.30 (2.69 to 3.91) | <0.001 | 4.15 (3.54 to 4.75) | <0.001 |
| Male sex |  | 0.45 (0.31 to 0.58) | <0.001 | -0.46 (-0.60 to -0.31) | <0.001 |
| Smoking behavior |  | 0.56 (0.45 o 0.66) | <0.001 | -0.42 (-0.55 to -0.30) | <0.001 |
| Alcohol consumption |  | 0.09 (-0.02 to 0.21) | 0.11 | -0.02 (-0.15 to 0.11) | 0.78 |
| N(waist circumference)_1_ |  | 0.69 (-1.30 to 2.67) | 0.50 | 1.53 (-0.38 to 3.44) | 0.12 |
| N(waist circumference)_2_ |  | 1.52 (0.65 to 2.39) | 0.001 | 0.21 (-0.94 to 1.35) | 0.72 |
| Prevalent type 2 diabetes |  | 0.32 (0.16 to 0.49) | <0.001 | 0.34 (0.19 to 0.48) | <0.001 |
| Prevalent cardiovascular disease |  | 0.60 (0.46 to 0.73) | <0.001 | 0.26 (0.13 to 0.40) | <0.001 |
| Prevalent cancer |  | 0.49 (0.23 to 0.75) | <0.001 | -0.13 (-0.27 to 0.01) | 0.075 |
| N(urinary albumin excretion)_1_ |  | 0.67 (0.35 to 1.00) | <0.001 | - | - |
| N(urinary albumin excretion)_2_ |  | 0.54 (0.39 to 0.68) | <0.001 | - | - |
| N(age)_1_ × N(waist circumference)_1_ |  | -1.86 (-5.93 to 2.21) | 0.37 | -5.02 (-8.85 to -1.19) | 0.010 |
| N(age)_2_ × N(waist circumference)_1_ |  | -0.19 (-1.47 to 1.09) | 0.77 | -0.56 (-1.84 to 0.73) | 0.40 |
| N(age)_1_ × N(waist circumference)_2_ |  | -2.33 (-4.05 to -0.61) | 0.008 | -0.36 (-2.57 to 1.85) | 0.75 |
| N(age)_2_ × N(waist circumference)_2_ |  | -0.58 (-1.10 to -0.06) | 0.028 | -0.68 (-1.40 to 0.05) | 0.067 |
|  |  |  |  |  |  |
| *A dash (-) indicates that the specific model term was not included in the final model. N(·)_1_ and N(·)_2_ denote the basis for a natural cubic spline with two degrees of freedom and natural boundary conditions imposed on the 2.5^th^ and 97.5^th^ percentiles of the relevant covariate to model potential nonlinearities. | | | | | |

**References**

1. UNESCO Institute for Statistics. International Standard Classification of Education. 2011http://uis.unesco.org/en/topic/international-standard-classification-education-isced.

2. Wang Z-M, Gallagher D, Nelson ME, Matthews DE, Heymsfield SB. Total-body skeletal muscle mass: evaluation of 24-h urinary creatinine excretion by computerized axial tomography. *Am J Clin Nutr* 1996;**63**:863–869.

3. Groothof D, Post A, Polinder-Bos HA, Erler NS, Flores-Guerrero JL, Kootstra-Ros JE *et al.* Muscle Mass and Estimates of Renal Function: A Longitudinal Cohort Study. *J Cachexia Sarcopenia Muscle* 2022;**13**:2031–2043.

4. Zipf G, Chiappa M, Porter K, Ostchega Y, Lewis B, Dostal J. National Health and Nutrition Examination Survey: plan and operations, 1999-2010. *Vital Health Stat 1* 2013;1–37.

5. Curtin LR, Mohadjer LK, Dohrmann SM, Montaquila JM, Kruszon-Moran D, Mirel LB *et al.* The National Health and Nutrition Examination Survey: Sample Design, 1999-2006. *Vital Health Stat 2* 2012.

6. Centers for Disease Control and Prevention. National Center for Health Statistics. National Health and Nutrition Examination Survey. https://www.cdc.gov/nchs/nhanes/index.htm. Accessed 6 January 2024.

7. Centers for Disease Control and Prevention. National Center for Health Statistics. National Center of Health Statistics Ethics Review Board (ERB) Approval. https://www.cdc.gov/nchs/nhanes/irba98.htm. Accessed 6 January 2024.

8. Centers for Disease Control and Prevention. National Center for Health Statistics. National Health and Nutrition Examination Survey (NHANES). Physician Examination Procedures Manual. https://www.cdc.gov/nchs/data/nhanes/nhanes_07_08/manual_pe.pdf. Accessed 6 January 2024.

9. Centers for Disease Control and Prevention. National Center for Health Statistics. National Health and Nutrition Examination Survey (NHANES). Anthropometry Procedures Manual. https://www.cdc.gov/nchs/data/nhanes/nhanes_07_08/manual_an.pdf. Accessed 6 January 2024.

10. Kim J, Wang Z, Heymsfield SB, Baumgartner RN, Gallagher D. Total-body skeletal muscle mass: estimation by a new dual-energy X-ray absorptiometry method. *Am J Clin Nutr* 2002;**76**:378–83.

11. Centers for Disease Control and Prevention. National Center for Health Statistics. National Health and Nutrition Examination Survey (NHANES). Laboratory Procedures Manual. https://www.cdc.gov/nchs/data/nhanes/nhanes_11_12/2011-12_laboratory_procedures_manual.pdf. Accessed 6 January 2024.

12. Finney H, Newman DJ, Gruber W, Merle P, Price CP. Initial evaluation of cystatin C measurement by particle-enhanced immunonephelometry on the Behring nephelometer systems (BNA, BN II). *Clin Chem* 1997;**43**:1016–22.

13. Sterne JAC, White IR, Carlin JB, Spratt M, Royston P, Kenward MG *et al.* Multiple imputation for missing data in epidemiological and clinical research: Potential and pitfalls. *BMJ* 2009;**339**:157–160.

14. Bartlett JW, Seaman SR, White IR, Carpenter JR. Multiple imputation of covariates by fully conditional specification: Accommodating the substantive model. *Stat Methods Med Res* 2015;**24**:462–487.

15. Centers for Disease Control and Prevention. National Center for Health Statistics. NHANES 1999-2006 DXA Multiple Imputation Data Files. https://wwwn.cdc.gov/Nchs/Nhanes/Dxa/Dxa.aspx. Accessed 6 January 2024.

16. Rubin DB. Inference and missing data. *Biometrika* 1976;**63**:581–592.

17. Rubin DB. *Multiple Imputation for Nonresponse in Surveys*. John Wiley and Sons: New York, NY; 1987.
